# Interleukin-10 Regulates Hepcidin in *Plasmodium falciparum* Malaria

**DOI:** 10.1371/journal.pone.0088408

**Published:** 2014-02-10

**Authors:** Honglei Huang, Abigail A. Lamikanra, Matthew S. Alkaitis, Marie L. Thézénas, Abhinay Ramaprasad, Ehab Moussa, David J. Roberts, Climent Casals-Pascual

**Affiliations:** 1 Wellcome Trust Centre for Human Genetics, Oxford, United Kingdom; 2 Nuffield Department of Clinical Laboratory Sciences, University of Oxford, and National Health Service Blood and Transplant, John Radcliffe Hospital, Headington, Oxford, United Kingdom; 3 Laboratory of Malaria and Vector Research, National Institute of Allergy and Infectious Diseases, Rockville, Maryland, United States of America; 4 King Abdullah University of Science and Technology, Saudi Arabia; Liverpool School of Tropical Medicine, United Kingdom

## Abstract

**Background:**

Acute malarial anemia remains a major public health problem. Hepcidin, the major hormone controlling the availability of iron, is raised during acute and asymptomatic parasitemia. Understanding the role and mechanism of raised hepcidin and so reduced iron availability during infection is critical to establish evidence-based guidelines for management of malaria anemia. Our recent clinical evidence suggests a potential role of IL-10 in the regulation of hepcidin in patients with acute *P. falciparum* malaria.

**Methods:**

We have measured secretion of hepcidin by primary macrophages and the hepatoma cell line HepG2 stimulated with IL-10, IL-6 and *Plasmodium falciparum*-infected erythrocytes.

**Findings:**

We have observed that IL-10 and IL-6 production increased in primary macrophages when these cells were co-cultured with *Plasmodium falciparum*–infected erythrocytes. We found that IL-10 induced hepcidin secretion in primary macrophages in a dose-dependent manner but not in HepG2 cells. These effects were mediated through signal transducer and activator of transcription (STAT) 3-phosphorylation and completely abrogated by a specific STAT3 inhibitor.

**Conclusion:**

IL-10 can directly regulate hepcidin in primary macrophages but not in HepG2 cells. This effect can be modulated by *Plasmodium falciparum*. The results are consistent with a role for IL-10 in modulating iron metabolism during acute phase of infection.

## Introduction

Anaemia remains a global public health problem world-wide that affects mainly young children and pregnant women [1], [2]. In Sub-Saharan Africa, malaria is likely to be one of the main contributors to anemia [3], [4]. It is clear that hepcidin, the major inhibitor of iron absorption and availability, is raised during acute and asymptomatic parasitemia and during infection [3], [5]–[7].

The majority of iron present in humans is in circulating erythroid cells. Any increase in iron requirements for developing erythroid cells is usually met by increasing intestinal absorption of dietary iron and release of iron from stores in macrophages. Macrophages store and supply iron to erythroblasts in the bone marrow. This is critical for the development of new red blood cells and failure to provide iron to developing erythroid cells will inevitably result in anaemia [8]. This process is tightly regulated by the liver-produced hormone hepcidin, the master regulator of iron homeostasis. Hepcidin inhibits iron absorption and its release from macrophages by down-regulating the concentration of the iron transporter, ferroportin on the cell surface of macrophages and enterocytes [9]. The expression of hepcidin is up-regulated when iron stores are replete, or by inflammation [10], [11] and is down-regulated by iron deficiency or hypoxia [12].

The importance of hepcidin in malaria has been recently highlighted by a study that identifies hepcidin, released during blood-stage parasitaemia, as a key inhibitor of *P. falciparum* liver-stage development [6]. There is some evidence that malaria-infected erythrocytes may increase hepcidin levels from peripheral blood mononuclear cells and that dietary iron absorption is decreased in adult women with asymptomatic malaria [13], [14].

We have recently provided evidence from a clinical study that parasitemia, interleukin-10 (IL-10) and interleukin-6 (IL-6) concentration were significantly associated with plasma hepcidin concentration in Kenyan children with acute falciparum malaria [15].

To understand the mechanism explaining this epidemiological observation, we have used malaria-infected erythrocytes and pro- and anti-inflammatory cytokines *in vitro* to investigate the release of hepcidin from macrophages and hepatocytes.

## Methods

### Macrophage isolation and culture

Macrophages were generated from peripheral blood mononuclear cells (PBMCs). Briefly, PBMCs were isolated from a single donor buffy coat (National Health Service Blood and Transplant, Filton, UK). PBMCs were resuspended in RPMI-10 consisting of RPMI (Lonza Biologics Plc, Slough, UK) containing 10% heat inactivated serum (SLI, Salisbury, UK), 50 mU/mL penicillin and streptomycin solution, 2 mM glutamine (Invitrogen, Paisley, UK) and 1% sodium pyruvate (Sigma, Poole, UK). All cultures were grown in a humidified incubator at 37°C in 5% CO_2_. Cells were seeded at 1×10^7^ cells per well of poly-D-lysine coated 6 well dishes (Scientific Laboratory Instruments, Nottingham, UK) or at 2.5×10^8^ cells per 150 cm^2^ flask (BD, Oxford, UK) and monocytes allowed to adhere at 37°C. After one hour non-adherent cells were removed using 2–3 washes with PBS. Collected cells were reapplied to fresh vessels for 1 hour to maximise capture of monocytes. Differentiation to macrophages was achieved by culturing in RPMI-10 containing 50 ng/mL M-CSF. M-CSF was replenished after 2–3 days. For experiments to study modulation of hepcidin and STAT-3 signalling, macrophages from day 5 or day 6 cultures were incubated for 12 hours (see Figure S1) with either LPS (Sigma, UK), IL-10 or live falciparum-infected erythrocytes (IEs). Supernatants were collected and stored at −20°C until analysis. Cells were carefully washed once with PBS containing protease inhibitor cocktail (Sigma, UK) and stored at −20°C until RNA or protein extraction.

### *Plasmodium falciparum* cultures

The *P. falciparum* (ITO4/A4) laboratory clone was cultured in erythrocytes as previously described [16], [17]. All cultures used in experiments were negative for *Mycoplasma* spp. based on the absence of mycoplasma enzyme activity using the MycoAlert Mycoplasma detection kit (Lonza Biologicals, Verviers, Belgium). Trophozoite and schizont stages of *P. falciparum* cultures were enriched to >90% purity by magnetic column isolation. Briefly, parasite cultures were centrifuged at 500 *g* for 5 minutes; the cell pellet was resuspended in 3 mL of supplemented serum-free RPMI-1640 (RPMI-SF containing 16 mM D-glucose (Sigma), 2 mM L-glutamine (Sigma), 37.5 mM HEPES (Lonza), 2.5 µg/mL gentamicin (Sigma), 1X HT supplement (Gibco, Invitrogen), pH 7.2–7.4) and applied to a LS column in a VarioMACS magnet (Milteny Biotec, Bergish-Gladbach, Germany). The column was washed with RPMI-SF until erythrocytes from the parasite culture were absent from the flow–through. The column was then removed from the magnet and the paramagnetic, parasitized cells were eluted in RPMI-SF. Purity of isolate was confirmed to be >90% by microscopic examination of Giemsa-stained thin film blood smear. All cultures used in experiments were negative for *Mycoplasma* spp.

### Quantification of hepcidin mRNA in human macrophages by RT-PCR

Total RNA was extracted directly from 3×10^5^ macrophages stimulated with IL-10, IL-6 or LPS using a QIAGEN RNeasy Kit (QIAGEN, UK) according to manufacturer's instructions. Total RNA concentration was quantified using NanoDrop ® 1000 Spectrophotometer (Thermo Scientific™), 40 ng of total RNA was reverse transcribed into cDNA by using SuperScript III cDNA Synthesis Kit (Invitrogen, UK). Gene expression was measured by real-time quantitative PCR using TaqMan® Gene Expression Assays on a StepOne thermocycler (Applied Biosystems). Expression was normalized to the constitutively expressed gene cyclophilin and using the ΔΔCT method [18].

### Western blotting

Macrophages were stimulated for 20 minutes with IL-10 at 1, 3, 10 and 30 ng/mL, LPS (100 ng/mL), IL-6 (40 ng/mL) or medium alone as control. Stimulated cells were lysed with RIPA buffer containing protease (Roche, USA) and phosphatase inhibitor cocktail (Sigma, UK). Protein concentration was quantified using the Pierce BCA Protein Assay (Thermofisher Scientific, Cramlington, UK). Samples containing 25 µg of protein were denatured at 95°C for 5 minutes in Laemmli buffer and loaded into 8–12% pre-cast SDS-PAGE gels (Bio-Rad, USA). Proteins separated by SDS-PAGE were transferred to PVDF membranes (Millipore, USA) in transfer buffer (25 mM Tris, 192 mM glycine and 10% methanol) overnight. PVDF membranes were blocked for 1 hour in TBST buffer (25 mM Tris, pH 7.5, 0.15 M NaCl, 0.05% Tween 20) containing 5% milk. Membranes were incubated for two hours at room temperature with mouse monoclonal anti-phospho-STAT3 (1∶1000 dilution), sheep monoclonal anti-STAT3 (1∶5000 dilution) (R&D systems, Abingdon, UK) or HRP-conjugated anti beta-actin as loading control (1∶25,000) (Sigma, Steinheim, Germany). Membranes were washed for 30 minutes with 5 changes of wash buffer and then incubated at room temperature for 1 hour in blocking buffer containing a 1∶5000 dilution of HRP-conjugated secondary antibody (Dako, UK). After washing for 30 minutes with 5 changes of blocking buffer, blots were developed on Kodak film (Sigma, Steinheim, Germany) using ECL plus reagent (GE Healthcare, UK).

### Hepcidin measurement

Hepcidin-25 levels were measured using Hepcidin-25 (human) – Enzyme Immunoassay kit (Bachem, UK) with the high-sensitivity protocol and following manufacturer's instructions. Briefly, samples were diluted 1/5 in standard diluent, and added to an immunoplate pre-coated with anti-hepcidin together with rabbit anti-serum. Plates were incubated for 2 hours at room temperature then washed 5 times with the manufacturer's wash buffer. One hour incubation with streptavidin-HRP was followed by a wash step and further hour's incubation with TMB solution. Reactions were then terminated using hydrochloric acid (2N) and the optical density (OD) read at 450 nm. Human Hepcidin-25 peptide was used as a standard and analysis was carried out according to the manufacturer's instructions.

### Statistical analyses

Independent t-tests or Mann-Whitney tests were used to compare means between independent variables or groups. The significance level α was set at 5%. Where more than one group was compared, analysis of variance (ANOVA) or the non-parametric equivalent (Kruskal-Wallis test) was used as appropriate and significance level adjusted, dividing α by the number of comparisons. Data were analysed using Stata version 11.0 (Stata Corporation, Texas, USA).

## Results

### *Plasmodium falciparum* up-regulates IL-6 and IL-10 production in primary macrophages

**Figure 1:**
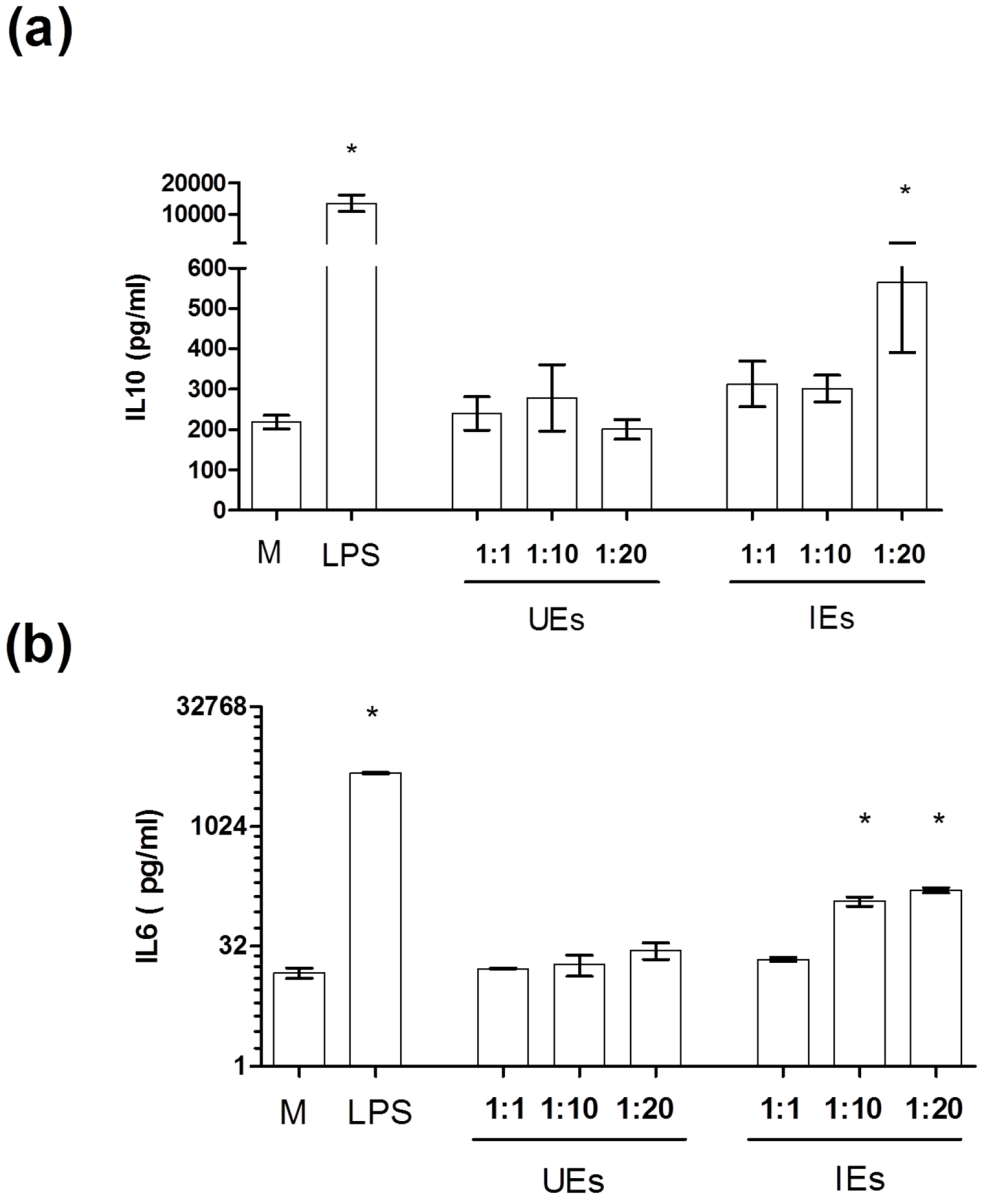
Concentration of IL-10 and IL-6 in supernatants of primary macrophages cultured with erythrocytes infected with *Plasmodium falciparum* (IEs) or uninfected erythrocytes (UEs) for 12 hours at increasing macrophage to parasite ratios. Data show mean concentration of (a) IL-10 and (b) IL-6 from 3 independent experiments. Error bars indicate standard error of the mean. M = media, LPS = lipopolysaccharide. * *P*<0.05 (Mann-Whitney test, compared with Media control).

Previous observations have suggested that *Plasmodium falciparum*-infected erythrocytes (IE) or their products could directly or indirectly induce hepcidin secretion from PBMCs. We investigated the production of hepcidin and IL-10 by primary macrophages (PM) co-cultured with IE. Erythrocytes infected with adhesive (ITO4/C10) parasite clones were co-cultured with macrophages to evaluate the role of parasite in the regulation of hepcidin (Figure 1). Higher IL-10 concentrations were found in culture supernatants of primary macrophages co-cultured with IEs at a 20: 1 ratio (IEs: PMs). No differences were found between adhesive (ITO4/C10) and non-adhesive parasite clones (T9/96) on IL-10 secretion (data not shown). Uninfected erythrocytes did not have any effect on IL-10-macrophage production. Similarly, PMs co-cultured with IEs showed a significant increase of IL-6 in culture supernatants (Fig 1b).

### IL-10 up-regulates hepcidin in primary macrophages but not in HepG2 cells

**Figure 2:**
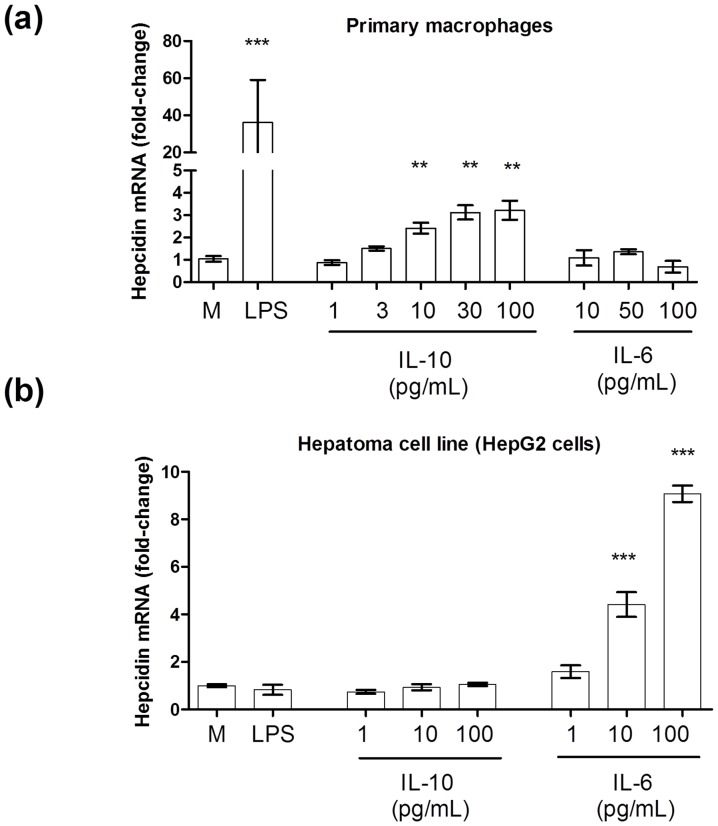
Effect of IL-10 and IL-6 on the regulation of hepcidin in (a) primary macrophages and (b) HepG2 cells. Data show fold-increase relative to mRNA in media control. Error bars indicate standard error of the mean. Bar plots show data from at least 3 independent experiments. ** *P*<0.01, ***P<0.001 (Mann-Whitney test, compared with Media control).

We have previously shown that circulating levels of IL-10 were closely associated with the concentration of serum hepcidin in children with acute falciparum malaria [15]. In order to determine if IL-10 could be a direct cause of hepcidin secretion, we used primary macrophages and cultured them with increasing concentrations of IL-10. Hepcidin mRNA increased with high concentrations of IL-10 (1–30 ng/mL) in a dose-response manner (Figure 2a).

**Figure 3:**
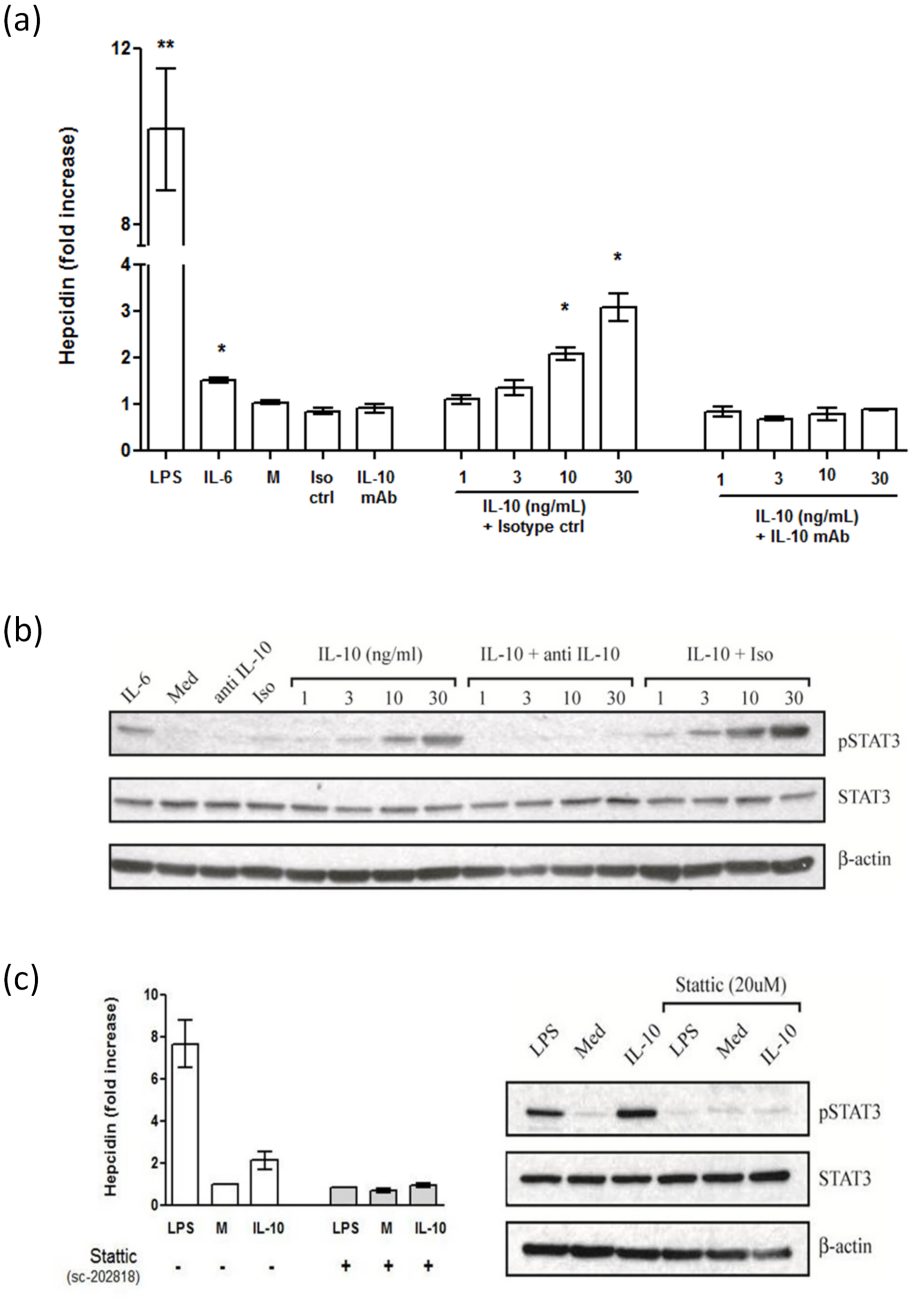
Regulation of hepcidin by IL-10 in primary macrophages, showing (a) the effect of IL-10 on Hepcidin mRNA synthesis with and without anti-IL-10 blocking antibody; (b) Western blot of STAT3 phosphorylation 20 minutes after exposure to increasing concentrations of IL-10; (c) STAT3-P inhibition of IL-10 (30 ng/mL) by Stattic. Bar plots show data from at least 3 independent experiments and the sho Western blot shows data from a representative experiment. M  =  media, iso ctrl  =  isotype control. * *P*<0.05, ***P<0.01 (Mann-Whitney test, compared with Media control).

The effect of IL-10 on hepcidin was completely inhibited when these cultures were pre-incubated with anti-IL-10 antibody (Figure 3a). The effect of IL-10 on hepcidin induction was mediated via STAT3-phosphorylation (Figure 3b). STAT3 phosphorylation was inhibited with a specific inhibitor of STAT3 phosphorylation (Stattic, 10 µg/mL) [19]. Cultured macrophages pre-incubated for 1 hour with this STAT3 inhibitor completely suppressed not only phosphorylation of STAT-3 but also the stimulatory effect of IL-10 (30 ng/mL) on hepcidin mRNA (Figure 3b &3c).

Most reports indicate that hepcidin is mainly produced in the liver. We therefore investigated the regulatory role of IL-10 on hepcidin in HepG2 cells. In this model cell line, IL-6 had a strong effect on hepcidin production in HepG2 cells with a greater than 15-fold increase in hepcidin mRNA after 12 hours of stimulation with IL-6 (100 ng/mL). However, hepcidin mRNA was not up-regulated in HepG2 cells stimulated with IL-10 (range: 1-100 pg/mL).

## Discussion

Here, we provide *in vitro* evidence of the role of IL-10 as a regulator of hepcidin production. IL-10 induces hepcidin in a dose-dependent manner through STAT3 phosphorylation in primary macrophages but not in the HepG2 hepatocyte line.

The importance of raised levels of hepcidin in host-parasite interactions during malaria has been recently highlighted in a study describing the critical role of this molecule to control super-infection of *P. falciparum* [6], [20]. High levels of hepcidin are induced by blood stage infection in density dependent but not species-specific manner and inhibit the development of *P. falciparum* liver stages. Although blood stages of *P. falciparum* have been shown to induce hepcidin mRNA in peripheral blood mononuclear cells [14], the mechanism(s) that drive hepcidin production during the blood stages of malaria are not well understood. Certainly, IL-6 has been associated with up-regulation of hepcidin and IL-6 levels are raised during acute infection [11]. We found IL-6 can induce transcription of hepcidin in hepatocytes but not in macrophages and in these liver cells IL-6-dependent induction of hepcidin is inhibited by hypoxia. However, high IL-6 levels appear to be part of a generalized acute phase response and do not completely explain the close association of hepcidin concentration and parasitemia.

An independent role for IL-10 stimulating hepcidin secretion during patent parasitemia is supported by our experimental observations that IL-10 induces hepcidin in a dose-dependent manner through STAT3 phosphorylation. Clearly, other signalling pathways must be invoked to mediate the distinct pro-inflammatory and anti-inflammatory effects of these cytokines.

**Figure 4:**
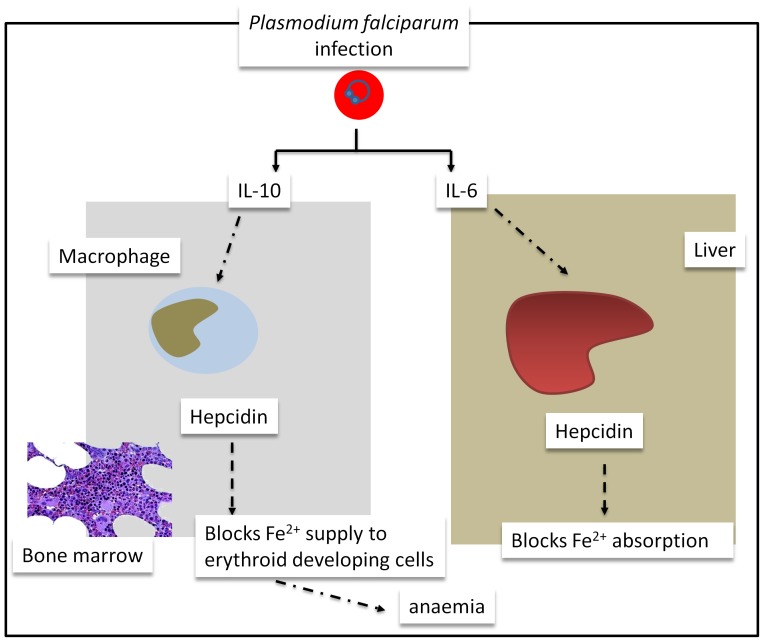
Proposed model of hepcidin in malaria infection. The regulation of hepcidin in response to infection may vary with cell type. A major response to infection occurs in hepatocytes in response to IL-6. However, our observations support the role of IL-10 in primary macrophages. Availability of iron to erythroid developing cells ultimately depends on macrophages and thus the high concentration of IL-10 may play a key regulatory role. Indeed, actively dividing cells like those found in the bone marrow are more susceptible to oxidative damage. In this context, both the direct anti-inflammatory effect of IL-10 and its indirect effect on iron restriction through the up-regulation of hepcidin may be beneficial.

We observed in our previous clinical study that IL-10 levels were very closely associated with parasitemia [15]. The pathway and source of IL-10 during acute infection is not completely understood but our data support a contribution of direct stimulation of IL-10 from macrophages by infected erythrocytes. We have shown whole live infected erythrocytes can stimulate IL-10 secretion from primary macrophages and, although IL-10 secretion was increased at higher parasite to macrophage ratios, the absolute amounts of IL-10 released were not enough, on their own, to reach the levels of IL-10 we observed to up-regulate hepcidin in macrophages. Therefore, we can only partly explain the parasite factors directly or indirectly associated with raised levels of IL-10. It may be that *in vivo* other cells and pathways contribute to a parasite-mediated IL-10 production and/or different monocyte and macrophage phenotypes induced during infection secrete significantly greater amounts of IL-10 in response to blood stage parasites (see Figure 4).

An effect of IL-10 on iron metabolism is well established but poorly understood. Indeed, a large randomized placebo-controlled study carried out in 329 patients showed a dose-dependent decrease of hemoglobin in patients receiving daily subcutaneous injections of IL-10 with doses from 1 to 20 µg/kg body weight [21]. Our data suggests one explanation for these apparently surprising observations, namely therapeutic IL-10 in these patients may have induced high levels of hepcidin leading to anaemia.

IL-10 has consistently been shown to be associated with favourable outcome in malaria [22]–[24] and other infections [25] and our data now link this protective anti-inflammatory response to hepcidin secretion. It is possible that the anti-inflammatory effects of IL-10 are mediated at least in part by a reduction in the availability of iron for proliferation of lymphocytes, by a reduction in circulating levels of iron available to catalyze reactions producing oxidative stress and/or a reduction in serum iron available to parasites.

The concentration of plasma IL-10 in patients with severe anaemia has been shown to be significantly lower than in patients with uncomplicated malaria or cerebral malaria [24]. Indeed, we and others have reported that hepcidin secretion appears to be blunted in patients with a Hb concentration below 5 g/dL [15], [26], possibly due to suppression of hepcidin secretion in response to hypoxia [12].

Our study highlights the role of IL-10 and hepcidin during blood stage malaria and at least in part demonstrates how hepcidin secretion may be stimulated by raised IL-10 during malarial infection. These observations link the anti-inflammatory cytokine, IL-10 with increased hepcidin levels during acute malaria and suggest how higher levels of IL-10 may lead to a favourable outcome in severe malaria.

## Supporting Information

Figure S1 **Time course of hepcidin mRNA induction by LPS in primary macrophages.**
(DOCX)
