# Supplementary material for: Interleukin-10 Regulates Hepcidin in Plasmodium falciparum Malaria
Source: PLoS One. 2014 Feb 10;9(2):e88408. doi: 10.1371/journal.pone.0088408 (PMC3919761; doi:10.1371/journal.pone.0088408)
Supplement: Figure S1 — Time course of hepcidin mRNA induction by LPS in primary macrophages. (DOCX) [file pone.0088408.s001.docx]

**sFigure 1.**
